# Supplementary figures and images for: Growth of 48 built environment bacterial isolates on board the International Space Station (ISS)
Source: PeerJ. 2016 Mar 22;4:e1842. doi: 10.7717/peerj.1842 (PMC4806633; doi:10.7717/peerj.1842)

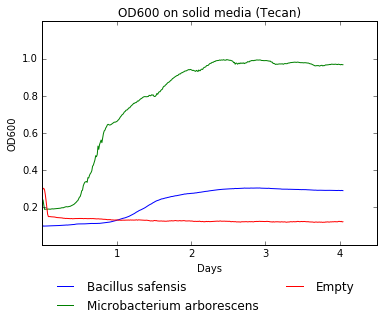

Supplement: Figure S1 — Values represent the mean of 25 measurements, on each of 25 spots, in each of 8 wells. These bacterial strains were grown for 96 h with OD600 measurements taken every 15 min in a Tecan F200 platereader. [file peerj-04-1842-s001.png]
